# Assessing Workplace Stress Among Nurses Using Heart Rate Variability Analysis With Wearable ECG Device–A Pilot Study

**DOI:** 10.3389/fpubh.2021.810577

**Published:** 2022-02-09

**Authors:** Xinxia Li, Weiwei Zhu, Xiaofan Sui, Aizhi Zhang, Lijie Chi, Lu Lv

**Affiliations:** ^1^Nursing Department, The Affiliated Hospital of Inner Mongolia Medical University, Hohhot, China; ^2^Prevention and Health Department, The Affiliated Hospital of Inner Mongolia Medical University, Hohhot, China; ^3^Intensive Care Unit, The Affiliated Hospital of Inner Mongolia Medical University, Hohhot, China; ^4^Neurosurgery Intensive Care Unit, The Affiliated Hospital of Inner Mongolia Medical University, Hohhot, China; ^5^Hangzhou Yicheng Business Management and Consulting Co., Ltd., Hangzhou, China

**Keywords:** heart rate variability (HRV), workplace stress, wearable biosensor, nurse, actual work condition

## Abstract

This study aims to measure workplace stress of nurses using heart rate variability (HRV) analysis based on data derived from wearable ECG heart rate monitors. The study population consists of 17 nurses at a major public hospital in China. Data was collected from 7 DON nurses (department of neurosurgery; all females; mean age: 31.43 ± 4.50), and 9 ICU nurses (intensive care unit; 8 females and 1 male; mean age: 31.33 ± 5.43). Each participant was asked to wear a wireless ECG heart rate monitor to measure stress level during work, and to complete the Chinese Nurses Stress Response Scale (CNSRS) after work as subjective response criteria. Demographic information, body posture, heart rate, R-R intervals (RRI), low frequency components (LF) and high frequency components (HF) were collected. LF%, LnHF and the squared root of the mean squared differences of successive NN intervals (RMSSD) based on HRV analysis were used to estimate the stress level of nurses. DON nurses reported a higher LF%, lower LnHF and lower RMSSD than ICU nurses. Work shifts were shown to have significant effects on LF%, LnHF and RMSSD respectively, with nurses in long shifts and night shifts reported high stress levels. Higher LF%, lower LnHF and lower RMSSD were found during work shift. Posture analysis revealed negative correlations with LnHF and RMSSD in walking and standing/sitting positions, and a significant negative correlation with LF% in lying-down position. Nurses with higher LF% reported higher CNSRS scores in all subscales, whereas nurses with lower LnHF or RMSSD reported higher CNSRS scores in social phobia and fatigue subscales. The results of this study support the idea that HRV can be used to investigate workplace stress among nurses under real work condition, and can serve as a preventive measure for identifying stress-related illnesses among nurses.

## Introduction

Nurses working in hospitals are exposed to high levels of workplace stress. In their daily routines, nurses can often experience high rates of physical and verbal violence, and harassment (1–3). Such high levels of workplace stress, if left untreated, can have harmful impacts on many aspects of their lives, manifesting into depression, anxiety, insomnia, burnout, poor self-esteem, and other mental-related health problems. It can further lead to poor decision-making, ineffective care, nurse-patient difficulties (4), and negative financial impact on hospitals and related institutions (5, 6). Thus, it is important to identify a reliable, cost-effective, sustainable method for early workplace stress detection in large team setting.

Many stress management programs and strategies for nurses have been evaluated by researchers, and it is notable that most studies investigated workplace stress based on self-report measures (7). However, these measures are mainly used for evaluating chronic stress (8). In recent years, the increasing interest in heart rate variability (HRV) research has produced extensive literature in numerous areas (9–11). In particular, cardiac vagal tone has been proposed as a physiological biomarker of stress (12–15). One non-invasive approach for investigating the vagal tone is by measuring the HRV suing electrocardiography (ECG) recording. With this technique, many studies have performed HRV analysis using ECG signals to investigate the feasibility of stress management (16, 17). HRV refers to the fluctuation of the length of heart beat intervals, and it represents the ability of the heart to respond to a variety of physiological and environmental stimuli (18). The power spectral density of HRV is usually calculated to assess the dynamics of the autonomic nervous system (ANS) (19). A number of neurobiological evidence suggests that HRV can be served as a reliable indicator of parasympathetic and sympathetic balance (7, 20, 21). Several studies investigating HRV measures further provided more complete pictures of ANS activation during stress conditions, with stress increasing sympathetic activity but decreasing parasympathetic activity (22–24). Analysis of HRV frequency components provides a powerful tool for evaluating stress states by simply recording ECG signals (25). In particular, HRV modifications in low-frequency (LF, 0.04 to 0.15Hz) and high-frequency (HF, 0.15 to 0.4 Hz) domains have found to be associated with stress exposure (26, 27). Furthermore, HRV is the beat-to-beat (R-R interval) variation in times between the consecutive heartbeats expressed in normal sinus rhythm on an ECG recording (28, 29). The HRV parameters in time domain, such as the standard deviation of the normal-to-normal interval (SDNN), the squared root of the mean squared differences of successive NN intervals (RMSSD), NN50, pNN50, were significantly decreased in specific experimental tasks for stress anticipation (7, 30). Various studies employed HRV indices to investigate the stress states, but participants in such studies were often placed in simulated experimental environment to induce stress.

This paper is a preliminary attempt to measure workplace stress level in nurses under real-world work conditions, and to examine how the HRV indices are correlated to their work postures, subjective stress response, workload and work shift. We believe this pilot study is a critical step in achieving an effective and comprehensive method to access real workplace stress for nurses.

## Methods

### Participants

A total of 17 nurses at the department of neurosurgery (DON) and intensive care unit (ICU) at a major public hospital in Inner Mongolia Autonomous Region of China participated in this study. The participants had no medical history of heart disease or other diseases potentially influencing HRV. All of them signed specific informed consent form prior to the test. Ethical permission was sought from the Ethic Committee of the Affiliated Hospital of Inner Mongolia Medical University.

### Wireless ECG Monitor

Each participant was asked to wear a wireless heart rate monitor (myBeat-WHS-1, Union Tool Co., Ltd., Japan) to obtain ECG measurement during work time from one workday. The heart rate monitor and the electrodes were mounted to the chest around the epigastrium by a dedicated chest strap. The triaxial acceleration, heart rate (HR), the time intervals between consecutive R-peaks (RRI), low frequency component (LF: 0.04-0.15Hz) and high frequency component (HF: 0.15-0.4Hz) of HRV signals, were recorded (31). With dedicated software (Viewer ver1.0.0), 3 types of postures (walking, standing/sitting, lying-down) were identified.

As indices of the influence of ANS, three HRV parameters, the LF% (LF/(LF+HF) x 100), LnHF and RMSSD (root mean square successive differences of RR intervals), were included to estimate the objective stress level of nurses in present study. High value of LF% is associated with high level of stress. Conversely, high value of LnHF and RMSSD is associated with low level of stress (7, 30). The LF%, LnHF and RMSSD were separately calculated offline using LF, HF and RRI data.

### Chinese Nurses Stress Response Scale

CNSRS is a scale designed to measure the subjective stress response of Chinese nurses (32), and it consists of 4 sub-scales (19 items): physiological reaction, irritability, social phobia, and fatigue. Subject is asked to rank each item along a 5-point scale from (1) “extremely uncharacteristic of me” to (5) “extremely characteristic of me.” A high score indicates high level of stress response. The scale has been tested and validated with 3,800 Chinese nurses (Cronbach's α ranged from 0.86 to 0.93, test-retest reliability coefficient = 0.86). In this study, CNSRS was used to evaluate workplace stress of nurses subjectively (Cronbach's α = 0.96, 4 sub-scales ranged from 0.78 to 0. 94).

### Work-Rest Record

During the experiment, the participants were asked to record work-rest at 15 min interval.

### Procedure

Before the experiment, information about the study was presented to the hospital management, the head nurses, and all the participants. Written approvals from the participants were also received. ECG signals were recorded continuously throughout the experiment during the entire work shift from one workday, including any break time or lunchtime. The participants were asked to record their work-rests at 15 min interval, and complete the CNSRS after their work shifts. For quality control, in each work shift, another nurse was designated as a research assistant to supervise the use of ECG device, the filling of work-rest record forms and psychological scales, and the recovery of ECG device from the participants.

### Statistical Analysis

We began with a preliminary analysis of the group differences for the demographic data of the participants. *T*-test for independent sample was, respectively, used to investigate the statistical significances (*p*-value < 0.05) of HRV parameters (LF%, LnHF, RMSSD) between work and rest phases, DON and ICU, different work postures (lying-down vs. standing/sitting vs. walking), while Pearson's correlation analysis was conducted between postures and HRV parameters. Analysis of variance (ANOVA) was performed on work shifts to examine the effect of work shifts on HRV parameters. For subjective measurement, multivariate ANOVA was used to analyze stress responses in different groups (HRV parameters × departments). The Statistical Package for the Social Science (SPSS) version 25.0 was used for all analysis.

## Results

### Demographic Characteristic

ECGs data from 17 healthy participants were collected, but data from 1 subject was excluded, due to corrupted data from a malfunctioned device. Table 1 presents the demographic information, which consists of 7 DON nurses (all females; mean age: 31.43 ± 4.50), and 9 ICU nurses (8 females and 1 male; mean age: 31.33 ± 5.43). There was no statistical difference in age (*t* = 0.037, df = 14, *P* = 0.971) and years of experience *(t* = −0.175, df = 14, *P* = 0.863) between two departments. No significant correlations were found between HRV parameters, age and years experience (*r* = −0.153~0.142). The number of nurses and patients in each shift on the test day is also presented in Table 1. By the Shapiro-Wilk test, all studied variables were normally distributed (*P* > 0.05).

**Table 1:** Demographic characteristic.

		**DON (***N*** = 7)**	**ICU (***N*** = 9)**	**t /χ2 -value**	**df**	***p*** **-value**
Age		(M = 31.43, SD = 4.50)	(M = 31.33, SD = 5.43)	0.037	14	0.971
Gender	Males	0 (0%)	1 (11%)	0.744	1	
	Females	7 (100%)	8 (89%)			
Professional status	Staff nurse	3 (42.9%)	3 (33%)	3.73	2	0.155
	Nurse practitioner	4 (57.1%)	2 (22%)			
	Charge nurse	-	4 (40%)			
Education	Bachelor	7	9	-	-	-
Work shifts	(I) 7:00~18:00	4 (57.1%)	-	-	-	-
	(II) 18:00~7:00	3 (42.9%)	-			
	(III) 8:00 ~15:00	-	3 (33%)			
	(IV) 15:00~22:00	-	3 (33%)			
	(V) 22:00~8:00	-	3 (33%)			
Patient number		17	9	-	-	-
Nursing Experience		1~15 years	1~16 years	−0.175	14	0.863
		(M = 8.86, SD = 5.27)	(M = 9.24, SD = 6.02)			

*DON, department of neurosurgery; ICU, intensive care unit; M, mean value; SD, standard deviation*.

### The HRV Parameters Changes in 24 H by Departments

**Figure 1:**
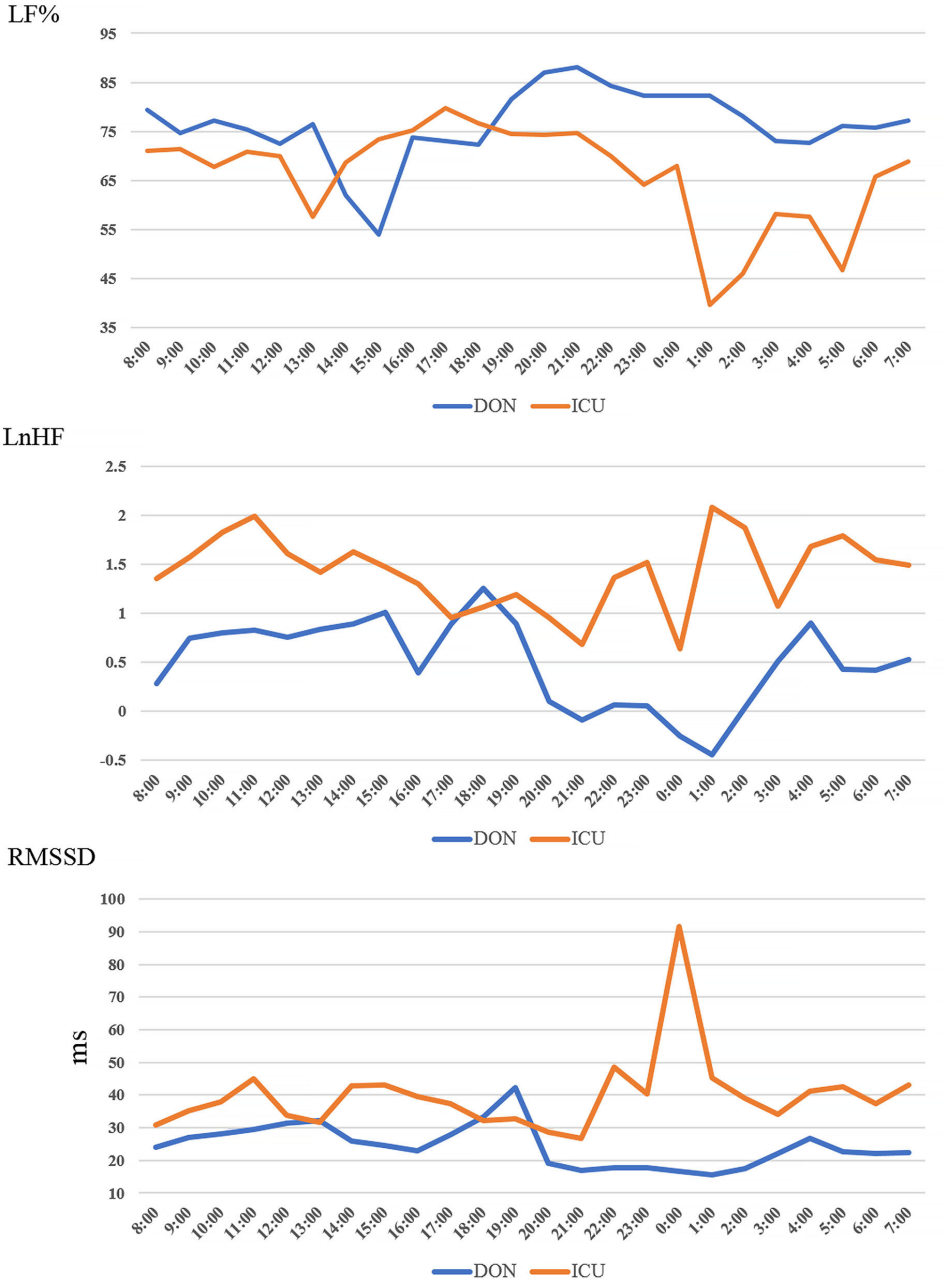
Changes of HRV parameters for DON and ICU nurses in 24 h.

Figure 1 shows the changes in LF%, LnHF and RMSSD for DON and ICU nurses within the 24 h experimental period. Data was computed as average value of every hour. As shown in Figure 1, LF% decreased, LnHF and RMSSD increased during lunch break and the period after mid-night. For different departments, the LF% values in DON nurses were higher than ICU nurses, whereas LnHF and RMSSD in DON nurses were lower than ICU nurses. The results suggested that stress level of ICU nurses was lower than that of DON nurses in general. In addition, correlation analysis revealed that LF% was strongly correlated with LnHF (*r* = −0.856, *P* = 0.000) and RMSSD (*r* = −0.821, *P* = 0.000), while a strong correlation was found between LnHF and RMSSD (*r* = 0.852, *P* = 0.000).

The mean values and standard deviations of LF%, LnHF and RMSSD are presented in Table 2. *T*-test result showed there were significant differences in LnHF and RMSSD, and no significant difference in LF% between DON and ICU nurses. In particular, LnHF (*t*_(14)_ = −2.956, *p* = 0.010) and RMSSD (*t*_(14)_ = −2.887, *p* = 0.012) of DON was lower than the ones of ICU, respectively.

**Table 2:** The mean differences and standard deviations of HRV parameters.

		***n***	**Mean ±SD**	**P25**	**P50**	**P75**	***t*** **-value**	**df**	**Sig**
LF%	DON	7	75.61 ± 7.88	68.40	78.74	82.17	1.707	14	0.110
	ICU	9	67.56 ± 10.32	62.37	66.33	75.46			
LnHF	DON	7	0.56 ± 0.60	0.02	0.43	1.10	−2.956	14	0.010
	ICU	9	1.43 ± 0.56	1.00	1.19	1.82			
RMSSD	DON	7	24.95 ± 7.45	19.15	21.43	29.17	−2.887	14	0.012
	ICU	9	37.91 ± 9.88	27.26	37.89	47.08			

*DON, department of neurosurgery; ICU, intensive care unit; LF, low frequency components; HF, high frequency components. RMSSD, the squared root of the mean squared differences of successive NN intervals; SD, standard deviation; P25, 25^th^ percentile; P50, 50^th^ percentile (same as median); P75, 75^th^ percentile*.

### HRV Parameters Correlations With Work Shifts

ANOVA testing with the *post hoc* Tukey HSD was used to assess the effects of work shifts on three HRV parameters. The results revealed that the mean value of LF%, LnHF and RMSSD were significantly different in work shifts groups (*F*_(4, 12)_ = 5.129, *P* = 0.014, *F*_(4, 12)_ = 3.692, *P* = 0.038, and *F*_(4, 12)_ = 2.940, *P* = 0.070, respectively). By mean differences comparison, the nurses in shifts II, IV reported higher values of LF% than in shift V, while those who worked in shift II reported lower levels of Ln HF and RMSSD than in shift III and V (see Table 3).

**Table 3:** Mean differences and standard deviations of HRV parameters by work shifts.

	**I**	**II**	**III**	**IV**	**V**	***F*** **-value**	**df**	**sig**	***Post-hoc***
LF%	71.86 (8.87)	80.60 (1.73)	68.66 (5.13)	76.77 (2.27)	57.24 (10.29)	5.129	4	0.014	II, IV > V*
LnHF	0.84 (0.67)	0.19 (0.21)	1.65 (0.35)	1.03 (0.13)	1.60 (0.89)	3.692	4	0.038	II < III*, II < V^†^
RMSSD	28.6 (8.20)	19.99 (1.25)	37.28 (9.16)	33.67 (12.44)	42.78 (9.40)	2.940	4	0.070	II < V^†^

*Work shifts I: 7:00~18:00; II: 18:00~7:00; III: 8:00~15:00; IV: 15:00~22:00; V: 22:00~8:00. LF, low frequency components; HF, high frequency components. RMSSD, the squared root of the mean squared differences of successive NN intervals*;

* *p < 0.05*;

† *p < 0.1*.

### HRV Parameters Correlations With Work and Rest Phases

A participant is recognized as in rest phase if there isn't any work in two consecutive 15-min periods. Otherwise it is regarded as in work phase. Mean and standard deviation were calculated to describe the distribution of HRV parameters during work and rest phases. As shown in Table 4, the LF% during work phase was significantly higher than that during rest phase (*t*_(106.538)_ = 9.293, *p* = 0.000), while the values of LnHF (*t*_(152.432)_ = −4.319, *p* = 0.000) and RMSSD (*t*_(612)_ = −2.232, *p* = 0.026) during work phase were significantly lower respectively.

**Table 4:** Mean differences of HRV parameters in work and rest phases.

		**Nn**	**Mean ±SD**	**P25**	**P50**	**P75**	***t*** **-value**	**df**	**Sig**
LF%	work	517	74.83 ± 11.00	67.71	76.14	83.42	9.293	106.538	0.000
	rest	97	54.92 ± 20.55	36.89	55.54	71.00			
LnHF	work	517	1.14 ± 1.06	0.38	1.21	1.80	−4.319	152.432	0.000
	rest	97	1.57 ± 0.89	0.87	1.58	2.13			
RMSSD	work	517	30.24 ± 15.88	18.84	26.65	36.98	−2.232	612	0.026
	rest	97	34.13 ± 14.98	22.24	32.31	41.42			

*SD, standard deviation. LF, low frequency components; HF, high frequency components. RMSSD, the squared root of the mean squared differences of successive NN intervals; SD, standard deviation; P25, 25^th^ percentile; P50, 50^th^ percentile (same as median); P75, 75^th^ percentile*.

### HRV Parameters Correlations With Work Postures

*T*-test was performed on work postures and departments, and the result is shown in Table 5. DON nurses were found to be in walking position (*t* = 2.013, *df* = 14, *P* = 0.064) and standing/sitting position (*t* = 3.530, *df* = 7.511, *P* = 0.009) more often than ICU nurses. Moreover, correlation analysis was performed on HRV parameters and postures. The result revealed that lying-down was significantly related to LF% (*r* = −0.613, *P* < 0.05). Meanwhile, LnHF and RMSSD were negatively correlated with walking (*r* = −0.471; *r* = −0.482), and standing/sitting (*r* = 0.466; *r* = −0.464), respectively, (see Table 6).

**Table 5:** Posture differences in ICU and DON departments (*n* = 16).

	**ICU**	**DON**	***t*** **-value**	**df**	***P*** **-value**
Walking	83.44 ± 42.73	135.29 ± 60.47	2.013	14	0.064
Standing/ Sitting	333.44 ± 56.29	531.86 ± 140.18	3.530	7.511	0.009
Lying-down	57.89 ± 89.22	57.00 ± 45.29	−0.026	12.369	0.980

*The results showed the mean value of time of three postural patterns in ICU and DON groups. ICU, intensive care unit; DON, department of neurosurgery*.

**Table 6:** Correlations among HRV-based variables and work postures (*N* = 16).

	**Standing/Sitting**	**Lying-down**	**LF%**	**LnHF**	**RMSSD**
Walking	−0.043	−0.182	0.410	−0.471^†^	−0.466^†^
Standing/ Sitting		−0.104	0.301	−0.482^†^	−0.464^†^
Lying-down			−0.613*	0.222	0.298

*LF, low frequency components; HF, high frequency components. RMSSD, the squared root of the mean squared differences of successive NN intervals*;

* *p < 0.05*;

† *p < 0.1*.

### HRV Parameters Correlations With Subjective Responses Using CNSRS

Table 7 lists the result of comparison between the objective stress level and the subjective stress response scores measured by the CNSRS in the two departments groups. The participants were assigned into low or high stress groups by their mean values of three HRV parameters, LF%, LnHF, and RMSSD. MANOVA was then used to explore the impact of HRV parameters and department (ICU, DON) on subjective stress responses.

**Table 7:** Mean differences of CNSRS subscale for HRV parameters and department (*N* = 16).

		**DON nurses**		**ICU**		**Main effects (** ***F*** **-value)**		**Interaction (***F***-value)**
		**Low**	**High**	**Low**	**High**	**Group effect (Low vs. high)**	**Departments (DON vs. ICU)**	
LF%	Physiological reaction	1.25 ± 0.35	1.93 ± 0.77	2.33 ± 0.85	3.38 ± 0.50	5.071*	10.864**	0.219
	Irritability	1.25 ± 0.35	2.50 ± 1.18	2.93 ± 0.95	4.00 ± 0.59	5.424*	10.241**	0.034
	Social phobia	1.83 ± 0.24	3.33 ± 0.71	1.93 ± 0.80	2.67 ± 1.41	4.915*	0.316	0.579
	Fatigue	2.00 ± 0.35	3.55 ± 1.04	3.05 ± 1.55	4.36 ± 0.60	5.736*	2.44	0.035
LnHF	Physiological reaction	1.93 ± 0.77	1.25 ± 0.35	3.11 ± 0.26	2.63 ± 1.05	1.651	8.144*	0.055
	Irritability	2.50 ± 1.18	1.25 ± 0.35	3.56 ± 0.42	3.33 ± 1.16	1.699	7.725*	0.828
	Social phobia	3.33 ± 0.71	1.83 ± 0.24	2.67 ± 1.53	2.06 ± 0.93	4.038^†^	0.179	0.716
	Fatigue	3.55 ± 1.04	2.00 ± 0.35	4.08 ± 0.38	3.42 ± 1.63	2.722	2.107	0.432
RMSSD	Physiological reaction	1.78 ± 0.79	1.50 ± 0.00	3.44 ± 0.59	2.47 ± 0.83	1.553	6.920*	0.479
	Irritability	2.25 ± 1.22	1.50 ± 0.00	4.17 ± 0.60	3.03 ± 0.88	2.139	7.113*	0.091
	Social phobia	3.11 ± 0.83	1.67 ± 0.00	3.22 ± 1.07	1.78 ± 0.81	6.649*	0.039	0.000
	Fatigue	3.33 ± 1.07	1.75 ± 0.00	4.50 ± 0.66	3.21 ± 1.44	3.524^†^	2.938	0.036

*Values are means (SD) of each subscale. DON, department of neurosurgery; ICU, intensive care unit; Low or High, low or high stress groups by their mean values of three HRV parameters; LF, low frequency components; HF, high frequency components. RMSSD, the squared root of the mean squared differences of successive NN intervals*;

** *P < 0.01*;

* *P < 0.05*;

† *P < 0.1*.

The mean differences between the low and high groups of LF% on physiological reaction, irritability, social phobia, and fatigue were statistically significant, with high groups reporting significantly higher subjective response than the low groups (*F*_(1, 12)_ = 5.071, *P* = 0.044, *F*_(1, 12)_ = 5.424, *P* = 0.038, *F*_(1, 12)_ = 4.915, *P* = 0.047 and *F*_(1, 12)_ = 5.736, *P* = 0.034, respectively). The mean difference between department groups was only found on physiological reaction and irritability subscales, with ICU groups reporting higher scores on the physiological reaction (*F*_(1, 12)_ = 10.864, *P* = 0.006) and irritability (*F*_(1, 12)_ = 10.241, *P* = 0.008). Divided by the mean value of LnHF, the group difference was only found on social phobia subscale, with low groups reporting higher scores than the high (*F*_(1, 12)_ = 4.038, *P* = 0.068). And ICU nurses reported higher scores on physiological reaction (*F*_(1, 12)_ = 8.144, *P* = 0.015) and irritability (*F*_(1, 12)_ = 7,125, *P* = 0.017) than DON nurses. The low value of RMSSD groups reported higher scores on two subscales, social phobia (*F*_(1, 12)_ = 6.649, *P* = 0.024) and fatigue (*F*_(1, 12)_ = 3.524, *P* = 0.085) than the high groups, respectively. The group differences were found on physiological reaction (*F*_(1, 12)_ = 6.920, *P* = 0.022) and irritability (*F*_(1, 12)_ = 7.113, *P* = 0.021), with ICU nurses reporting higher scores than DON nurses. There was no significant interaction effect between departments and HRV parameters (LF%, LnHF, RMSSD).

## Discussion

In general, our results demonstrated that it is feasible to use HRV analysis, based on ECG signal collected by wearable ECG device, to investigate stress under real work conditions among nurses. In fact, using wearable devices and HRV to monitor mental stress level is gaining attention among the scientific community (33).

DON nurses were found to have higher LF%, lower LnHF and lower RMSSD than ICU nurses, which indicates that DON nurses experienced higher level of objective stress than ICU nurses. One possible explanation is that while the number of nurses on-duty were similar in DON and ICU, there were 17 patients in DON and only 9 patients in ICU.

The study also highlighted how work shifts affected LF%, LnHF and RMSSD. It is critical to note that the work shifts were different in DON (shift I and II) and ICU (shift III, IV and V). The results showed higher mean values of LF% and lower means of LnHF and RMSSD in DON shifts. Our findings are consistent with previous studies, which suggested that long hour shift significantly affects the overall physical and mental health of nurses, and job-related stress varies across working unit (34, 35). For mean differences comparison among work shifts, we found that LF% in shift II and IV was significantly higher than shift V, while LnHF and RMSSD in shift II was lower than III and/or V. This indicated that nurses working in night shifts experienced higher level of physical and mental stress than those who worked in day shifts.

In contrast, LF% at work phase was significantly higher than at rest phase, while LnHF and RMSSD at work phase were significantly lower than at rest phase. These findings are in good agreement with previous study, which suggested that work-rest schedules influence physiological and postural workload, and cause worker to subjectively feel more fatigue during work phase (36). Long work hours_may lead to fatigue and different types of injuries during the operation (37, 38), which may cause psychological, physiological and musculoskeletal disorders in long run (39).

Regarding work postures, only lying-down was found to have significant negative correlation with LF%. For walking and standing/sitting positions, negative correlations with LnHF and RMSSD were found, and no significant correlation with LF%. This discrepancy may be caused by the small sample size and the hardware limitation. Since the ECG monitor used in present study cannot recognize the standing and sitting positions separately, the combined measurement of work postures may have affected the result. Overall, our findings are consistent with previous studies which indicated that walking and standing/sitting for long time are shown to influence physiological and psychological responses linked to an increased risk of stress (40–42). Prolonged poor posture can lead to a prolonged distressed state. Improvement in postural changes may increase positive effect, reduced fatigue, and alleviate stress (43). In addition, the result showed that DON nurses spent more time in walking and standing/sitting positions than ICU nurses, which may explain the higher LF%, lower LnHF and RMSSD among DON nurses.

The CNSRS result showed that nurses with high LF% reported higher levels of physiological reaction, irritability, fear for social activity and fatigue. Also, nurses with high LnHF and RMSSD reported lower level of social phobia and fatigue. This confirms that the HRV parameters (LF%, LnHF and RMSSD) are in line with the results from subjective measurements.

It is important to note that ICU nurses showed higher scores of physiological reaction and irritability than DON nurses in CNSRS. This finding may seem to be in contradiction with the result measured by the HRV parameters, in which ICU nurses had a lower objective stress response than DON nurses. However, according to the experiment log, the workload of ICU on the experiment day was considerably lower than average. As a result, it can be argued that ICU nurses experienced lower acute stress on the experiment day, while they were experiencing higher chronic stress as shown in the CNSRS. In fact, previous finding has indicated that ICU nurses generally report feeling higher stress and experience stress responses (e.g., fatigue, distress, irritability, etc.) at a higher rate than nurses in other units (44).

A number of limitations might influence the results obtained. The sample size of this study is small, and it may affect the statistical power. The experimental period was only 1 day, so it may affect the generality of our results. In future studies, individual differences among nurses should also be considered. Additionally, nearly half of the participants reported certain discomfort when wearing the ECG device, and this issue should be addressed in future research. Also, there exists other devices with less intrusive data acquisition methods such as the Oura Ring, Fitbit Charge and Apple Watch. In future study, these devices should be considered and evaluated against the present ECG device in their feasibility in monitoring workplace's stress states of nurses under normal working-conditions. Future works exploring the effects of extended use of ECG device and HRV analysis for measuring stress, as well as studies on nurses at other departments should be conducted.

## Conclusion

In conclusion, this pilot study investigated the method of using ECG heart rate monitor and LF%, LnHF and RMSSD as HRV-based stress indicators to assess workplace stress in nurses under real-working condition. CNSRS was used as subjective stress evaluation criteria, and it was shown to be correlated to the values of LF%, LnHF and RMSSD. The results also showed that departments, work shifts, and work posture were correlated to the stress level of nurses. Overall, this pilot study demonstrated that it is feasible to use wearable ECG device and HRV analysis to investigate workplace stress under work conditions in nurses. This approach may also serve as a preventive measure for identifying stress-related illnesses in nurses.

## Data Availability Statement

The raw data supporting the conclusions of this article will be made available by the authors, without undue reservation.

## Ethics Statement

The studies involving human participants were reviewed and approved by the Ethic Committee of the Affiliated Hospital of Inner Mongolia Medical University. The participants provided their written informed consent to participate in this study.

## Author Contributions

XL contributed to conceptualization, supervision, project administration, and funding acquisition. XS investigated the resource. AZ and LC participated in the data collection. LL contributed to formal analysis. WZ wrote the first draft of the paper. XL and WZ revised and edited the final manuscript. All authors have read and agreed to the published version of the manuscript.

## Funding

This research was supported by a project grant from National Health Commission for Health Professional Development Program of China (Grant No. 2019-HLYJ-015).

## Conflict of Interest

LL was employed by company Hangzhou Yicheng Business Management and Consulting Co., Ltd. The remaining authors declare that the research was conducted in the absence of any commercial or financial relationships that could be construed as a potential conflict of interest.

## Publisher's Note

All claims expressed in this article are solely those of the authors and do not necessarily represent those of their affiliated organizations, or those of the publisher, the editors and the reviewers. Any product that may be evaluated in this article, or claim that may be made by its manufacturer, is not guaranteed or endorsed by the publisher.
